# Pizza and Paintballs: A Cost-Effective Model for Incision and Drainage Simulation Training

**DOI:** 10.21980/J8.52047

**Published:** 2025-10-31

**Authors:** Patrick McNeal, Andrea D Boan, Emily Douglas

**Affiliations:** * Medical University of South Carolina, College of Health Professions, Division of Physician Assistant Studies, Charleston, SC

## Abstract

**Audience:**

This innovation aims to educate medical students, physician assistant students (PA), and medical residents across various levels.

**Background:**

Skin abscesses are frequently encountered in clinical practice, and incision and drainage (I&D) is a common treatment performed in both emergency and outpatient settings.^1^,^2^ For advanced practice providers (APPs) and emergency medicine residents, learning this technical skill is particularly important, given the prevalence of abscesses in primary care and urgent care settings. At the Medical University of South Carolina (MUSC), PA students are taught this procedure during the didactic phase of their clinical skills and procedures course.

However, the cost of supplies for teaching this procedure has been a significant expense for the program, with commercial abscess task trainers ranging from $22.09 for a Pocket Nurse single-use pad to $65.99 for a SurgiReal tissue pad.^3^ In contrast, our model cost is approximately $198 for ~100 learners, demonstrating substantial cost savings for resource-limited programs.

This manuscript offers a comprehensive guide for crafting an innovative, cost-effective, and true-to-life simulation model designed specifically for I&D training for healthcare practitioners. The materials required for this model can be conveniently sourced from Amazon.com. The proposed technique aims to significantly enhance the educational experience of healthcare practitioners as they acquire these vital clinical skills.

**Educational Objectives:**

Upon completing this lab session, the participant should have the capability to: 1) describe the indications, contraindications, and reasons for performing I&D of an abscess, 2) select the necessary equipment for performing I&D of an abscess, 3) demonstrate the necessary steps for performing an I&D procedure on a simulated abscess.

**Educational Methods:**

The lab was designed for an 8:1 trainee-to-instructor ratio and a 45-minute duration, and utilized paintballs inserted under rolled pizza dough to simulate “skin.” Trainees practiced using lidocaine, syringes, and needles with sterile water, and performed the field block technique. They made incisions in the “abscess” using scalpels, expressed “purulent” material (white paint), removed the “capsule” (paintball shell), and swabbed the material with sterile swabs. Materials included Chux pads, pizza dough, paintballs, gloves, alcohol swabs, needles, syringes, sterile water, scalpels, and applicators, costing $198.06 in total, or $9.56 per student, mostly purchased from Amazon.com.

**Research Methods:**

A Redcap survey was distributed to evaluate the effectiveness of an abscess drainage lab in meeting predefined learning objectives. The survey, sent to 188 students, received a 35% response rate. Participants were asked five questions, rated on a 5-point Likert scale, assessing the lab’s value in teaching incision and drainage (I&D), field block, and needle safety. Responses were dichotomized into “disagree/strongly disagree” and “agree/strongly agree.”

**Results:**

The lab was positively evaluated, with 86.15% (95% CI 75.3–93.5) of respondents recommending the lab be repeated, and 81.82% (95% CI 70.4–90.2) stating the lab provided a realistic simulation of abscess drainage. Additionally, 84.85% (95% CI 73.9–92.5) of participants reported improved confidence in performing I&D, 87.88% (95% CI 77.5–94.6) felt their field block skills improved, and 93.94% (95% CI 85.2–98.3) expressed increased comfort with needle safety.

**Discussion:**

The simulation lab teaches crucial skills such as abscess incision and drainage, field block dexterity, and needle safety during lidocaine preparation. It offers a safe environment for students to practice these skills before patient interaction in clinical settings, using cost-effective materials like paintballs and pizza dough. Similar low-cost abscess simulation models have been described, including reusable ultrasound-guided training methods, further supporting the value of inexpensive, reproducible alternatives for procedural education.^4^ Although cyst wall removal is included in the simulation checklist, in actual practice this is often not attempted during acute drainage when inflamed. Patients are generally referred to outpatient surgery for follow-up.

While there are limitations to this method, including inability to practice breaking up loculations and limitations of the authenticity of the pizza and paintballs, the cost-effectiveness of the lab allows for students an engaging avenue to practice these skills and gain confidence prior to entering clinical rotations. Also, the low response rate among students surveyed likely is secondary to delay between the lab and survey distribution, and could be improved with repeating this study with a shorter time interval between lab and survey with another cohort. Future studies could evaluate the pizza and paintball model in a noninferiority design compared to commercial task trainers to determine whether this cost-effective method maintains equivalent educational quality in programs with greater resources. While there are limitations in the response rate among students surveyed, the overall results reveal that utilizing this method improves student confidence in I&D, needle safety, and field block technique. The pizza and paintball I&D lab provides a cost-effective simulation of I&D and field blocks that can be replicated for use in other medical education programs.

**Topics:**

Abscess incision and drainage, cost-effective training, simulation, I&D.

## USER GUIDE

**Table:** 

List of Resources:	
Abstract	7
User Guide	10
Abscess I&D PowerPoint	15

**Learner Audience:**

Medical Students, Interns, PA/NP students, APP Fellows

**Time Required for Implementation:**

Construction of the model after the artificial spine and ribcage have been printed takes approximately five to eight hours (3D printing takes about 22 hours). Training sessions take approximately 30 minutes to complete: 5–10 minutes for introductory didactic lecture, five minutes for instructor demonstration of the ESP nerve block on model, and 15–20 minutes for participants to practice.

**Instructor Time Spent Creating the Innovation:** The preparation time might vary between two to four hours. Specifically, it requires around two hours for the paintballs to fully absorb into the pizza dough, achieving the desired fluctuant texture.

**Learners’ Time Using the Innovation:**

- **Per Session:** The lab session is designed with a completion time of approximately 45 minutes for a group of eight students with one instructor. Each student spends this time engaging in various aspects of the simulated procedure, from practicing needle techniques to incision and drainage.
- **Total Length of Time Necessary to Develop Competence:** Competence development in medical procedures like abscess I&D typically requires repeated practice, observation, and feedback. Learners may need multiple sessions spread over several days or weeks to attain proficiency. Developing competence can range from one to two sessions to more extensive practice over a semester or academic term, depending on the learning curve and individual student progress.

**Recommended Number of Learners per Instructor:**

The recommended ratio of instructors to learners when utilizing this innovation for instruction is one instructor for every eight learners.

**Topics:**

Abscess incision and drainage, cost-effective training, simulation, I&D.

**Objectives:**

Upon completing this lab session, the participant should have the capability to:

1. Describe the indications, contraindications, and reasons for performing I&D of an abscess.
2. Select the necessary equipment for performing I&D of an abscess.
3. Demonstrate the necessary steps for performing an I&D procedure on a simulated abscess.

### Linked objectives, methods and results

The goals and objectives of teaching abscess I&D through the pizza and paintball simulation format are achieved through a structured and hands-on approach designed to simulate a realistic clinical scenario. The chosen format aligns with established educational objectives by providing a comprehensive learning experience for healthcare practitioners. The following conceptual framework was considered while developing the content:

1. **Engagement and Realism:** The simulation format involves using pizza dough as simulated skin and paintballs as abscesses, creating a tangible and realistic scenario. Learners engage in hands-on practice, mimicking the steps of an actual I&D procedure. This approach promotes active engagement, aiding in the retention of knowledge and skill development.
2. **Structured Learning:** The session follows a structured format, starting with equipment training, progressing to technique practice, and concluding with procedure simulation (objectives 1, 2 and 3). This step-by-step approach ensures learners are introduced to each aspect of the procedure in a systematic manner, enhancing comprehension and skill acquisition.
3. **Guided Practice and Evaluation:** The instructor-student ratio of one to eight allows for adequate supervision and guidance during practice. A checklist evaluation ensures that learners adhere to proper techniques, sterile procedures, and completeness of the I&D procedure (objective 3). Feedback and debriefing sessions foster reflective learning, addressing any challenges encountered.
4. **Relevance to Clinical Practice:** The simulation closely mirrors real-life scenarios encountered in clinical settings, allowing learners to transfer acquired skills directly to patient care. By practicing on a simulated abscess, learners gain confidence and familiarity with the procedure, preparing them for future clinical encounters.

### Recommended pre-reading for instructor

Participants and instructors are recommended to review the “Incision and Drainage of Superficial Abscess” section in Chapter 7 of CURRENT.^6^ Instructors have the option to deliver a live presentation or pre-record a lecture using the attached Abscess I&D PowerPoint.

### Learner responsible content (LRC)

- Participants must review the “Incision and Drainage of Superficial Abscess” section in Chapter 7 of CURRENT.^6^
- Attend live presentation or pre-recorded lecture of the abscess I&D PowerPoint.

### Implementation Methods

Before the session, the preparation involves inserting paintballs under rolled pizza dough one to two hours beforehand, allowing them to be absorbed and create a fluctuant texture resembling an abscess. The session begins with a brief introduction outlining the objectives and activities. This is followed by a short training on the equipment, where students practice using lidocaine, syringes, needles (18G and 27G), and other necessary tools. The students then practice the field block technique on the pizza dough, which now contains absorbed paintballs, to simulate anesthesia administration.

Next, the students perform an I&D procedure on the simulated abscess. They make an incision on the “abscess” in the pizza dough using a scalpel, express the “purulent” material (represented by white paint), and remove the “capsule” (paintball shell) while maintaining sterile techniques. Swabbing the purulent material for culture is also part of the practice. During the procedure, instructors evaluate the students based on a checklist that covers proper technique, sterile procedures, and the completeness of each step.

The checklist includes ensuring aseptic procedures, administering anesthesia, making an incision with a pointed no. 11 scalpel, draining and exploring the site, and removing the cyst capsule. Routine packing of drained abscesses is not recommended because studies show no improvement in outcomes and increased patient pain. Packing should only be considered in areas of skin apposition (axilla, groin, gluteal cleft), and dressing the wound.^5^ The checklist also includes learners providing follow-up care instructions, including daily packing removal and re-dressing until the wound heals by second intention. The checklist also covers the disposal of needles and equipment, glove removal, and proper documentation of the procedure, including date, time, site, and any complications. While purulent drainage was once used as a surrogate for MRSA, updated IDSA guidance emphasizes evaluating patient and community-level risk factors (eg, prior MRSA colonization, recent healthcare exposure, antibiotic use, close contact environments, injection drug use). The practice session concludes with a discussion and debrief where challenges are reviewed and key learning points are reinforced. Students may also be encouraged to review relevant literature or resources post-session to reinforce their learning.

### List of items required to replicate this innovation

1. **Chux Pads**
  - Quantity: 1 pack (several pads in a pack)
  - Website/Store: Amazon.com, Medical supply stores
2. **Pizza Dough**
  - Quantity: Sufficient for 1 “pizza” per 8 students
  - Website/Store: Local grocery stores (such as Walmart, Kroger, or Safeway) or local pizzerias that sell raw pizza dough
3. **White Paintballs**
  - Quantity: 2 per student (considering 8 students)
  - Website/Store: Paintball supply stores, sporting goods stores, or online retailers like ANSgear.com or Amazon.com
4. **Nitrile Gloves**
  - Quantity: Sufficient for 8 students
  - Website/Store: Amazon.com, Medical supply stores, Home Depot, or Lowes
5. **Alcohol Swabs**
  - Quantity: Sufficient for 8 students
  - Website/Store: Amazon.com, CVS, Walgreens, or medical supply stores
6. **18G × 1″ Needle**
  - Quantity: At least 1 per student
  - Website/Store: Medical supply stores, Amazon.com
7. **27G × 1.5″ Needle**
  - Quantity: At least 1 per student
  - Website/Store: Medical supply stores, Amazon.com
8. **5 mL Sterile Luer Lock Syringe**
  - Quantity: At least 1 per student
  - Website/Store: Medical supply stores, Amazon.com
9. **20 mL Sterile Water Vial**
  - Quantity: Sufficient for 8 students
  - Website/Store: Medical supply stores, Amazon.com
10. **Scalpel, No. 11**
  - Quantity: At least 1 per student
  - Website/Store: Medical supply stores, Amazon.com
11. **Sterile Cotton-Tipped Wood Applicator**
  - Quantity: Several packs for 8 students
  - Website/Store: Medical supply stores, Amazon.com

### Approximate cost of items to create this innovation

The total cost for the materials required to create this innovation for the entire lab session, including pizza dough, paintballs, and I&D equipment, amounted to $198.06. This cost covers the materials necessary for a group of approximately 100 students, with each learner able to perform at least one simulated abscess I&D.

### Detailed methods to construct this innovation

**Step 1: Gather Materials** Gather all the required materials and set them up in a clean and organized workspace.

**Step 2: Set Up Workspace** Lay down Chux pads for cleanliness. Arrange the necessary equipment nearby for easy access during the simulation.

**Step 3: Prepare Pizza Dough** Take the pizza dough and roll it out into a size resembling human skin. Ensure it is thick enough to hold the paintballs and simulate the abscess.

**Step 4: Insert Paintballs** Place the white paintballs strategically under the rolled-out pizza dough. Space them out to represent different abscess locations and depths.

**Step 5: Wear Protective Gear** Put on nitrile gloves for safety and hygiene.[Fig f1-jetem-10-4-i7]

**Step 7: Practice Anesthesia Administration** Demonstrate and allow participants to practice anesthesia administration using the sterile water vial, syringes, and needles on the pizza dough’s surface.[Fig f2-jetem-10-4-i7]

**Step 8: Practice I&D Procedure** Guide participants through the I&D procedure steps:

- Use the scalpel to make an incision on the dough over the paintball.[Fig f3-jetem-10-4-i7]
- Simulate expression of “purulent” material (white paint).[Fig f4-jetem-10-4-i7][Fig f5-jetem-10-4-i7]
- Use curved or straight hemostats to disrupt or break apart loculations.
- Use sterile cotton-tipped wood applicators to swab the “purulent” material for culturing purpose

### Results and tips for successful implementation

This abscess I&D lab was designed to train healthcare practitioners in performing abscess drainage with a focus on simulation models, educational objectives, materials, and procedures. The lab was tested on 188 PA students, with feedback gathered via a Redcap survey sent to the 188 participants, yielding a 35% response rate. The survey used a 5- point Likert scale, dichotomized into “agree/strongly agree” and “disagree/strongly disagree” categories to assess the lab’s effectiveness in teaching I&D, field block, and needle safety.

**Figure 6:**
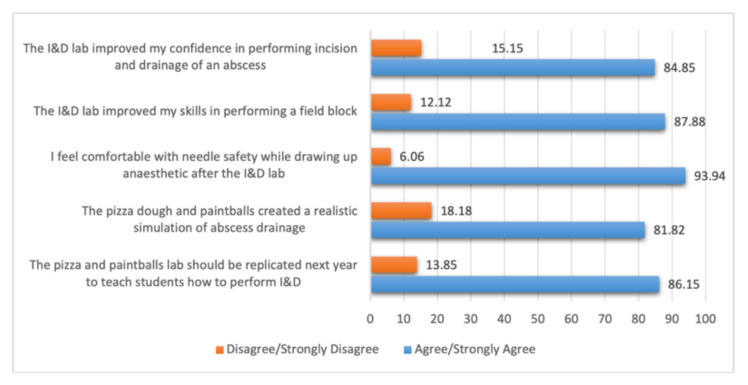
Summary of Survey results.

**Figure 7:**
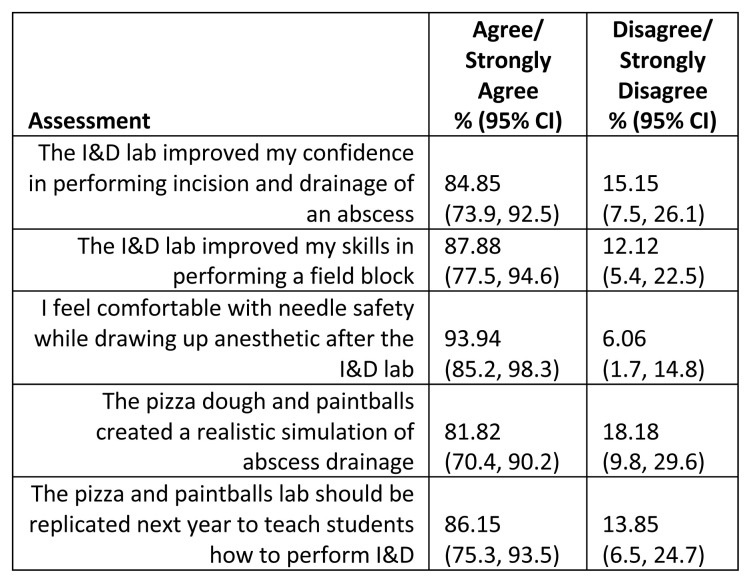
Pizza and Paintballs I&D Lab Student Evaluations.

The survey results revealed an overall positive evaluation of the lab with 86.15% (95% CI 75.3, 93.5) of respondents reporting that the lab should be replicated the next year, and 81.82% (95% CI 70.4, 90.2) reporting that the lab created a realistic simulation of abscess drainage. The lab was also successful teaching the skills of I&D, field block and needle safety, as seen in the following data: 84.85% ( 95% CI 73.9, 92.5) of respondents report improved confidence in performing I&D, 87.88% (95% CI 77.5–94.6) of respondents report improved field block skills and 93.94% (95% CI 85.2, 98.3) of respondents report comfort in needle safety following the lab (figures 6, 7).

Sample n=66. Data are presented as binomial proportions with Clopper-Pearson (Exact) 95% confidence intervals. All items yielded significant differences for the asymptotic equality test for the binomial proportion (all p<0.0001).

A redcap survey of student participants was utilized to determine whether the lab was sufficient for meeting the defined learning objectives. The survey was sent to 188 students, with a 35% response rate. The survey instrument contained five questions graded on a 5-point Likert-scale assessing the labs’ value in teaching skills including incision and drainage, field block, and needle safety. These results were then dichotomized into “disagree and strongly disagree” and “agree/strongly agree.”

Student feedback was largely positive, with comments highlighting the engaging and realistic nature of the lab, especially the use of “pizza cysts” and paintballs as simulated abscesses. The hands-on experience with skills that are directly applicable to clinical practice was well-received. For future sessions, it may be beneficial to adjust the scheduling to ensure adequate time for the paintballs to dissolve into the dough. Previously, lab sessions were held consecutively, which allowed sufficient time for paintball softening for the first group of participants but resulted in more firm paintballs for subsequent groups due to shorter intervals. By scheduling sessions on separate days, we can offer a more consistent and realistic simulation experience for all groups, ensuring that the quality of the session is uniform, not just for the first group. Overall, the lab’s design and execution successfully enhanced procedural skills and confidence. The overwhelmingly positive feedback suggests the lab’s structure is effective, with minor adjustments potentially needed to further ease the transition from simulation to clinical practice.

## Supplementary Information



## Figures and Tables

**Figure:**
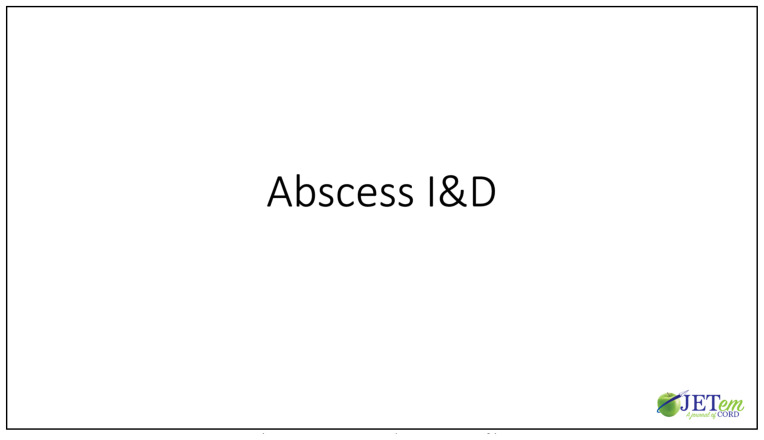
Please see associated PowerPoint file

**Figure 1 f1-jetem-10-4-i7:**
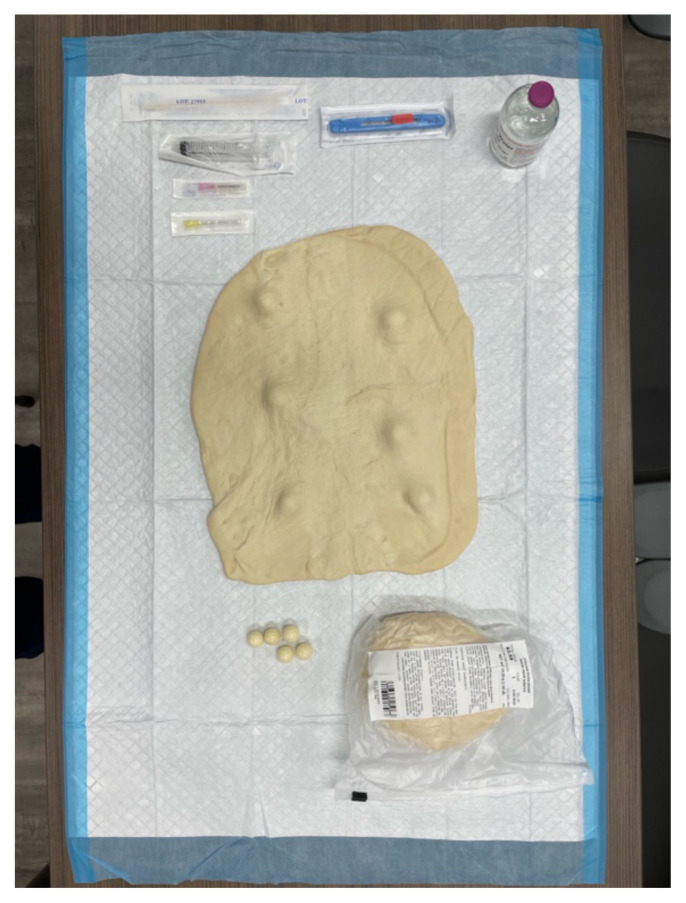
Equipment layout on a Chux pad.

**Figure 2 f2-jetem-10-4-i7:**
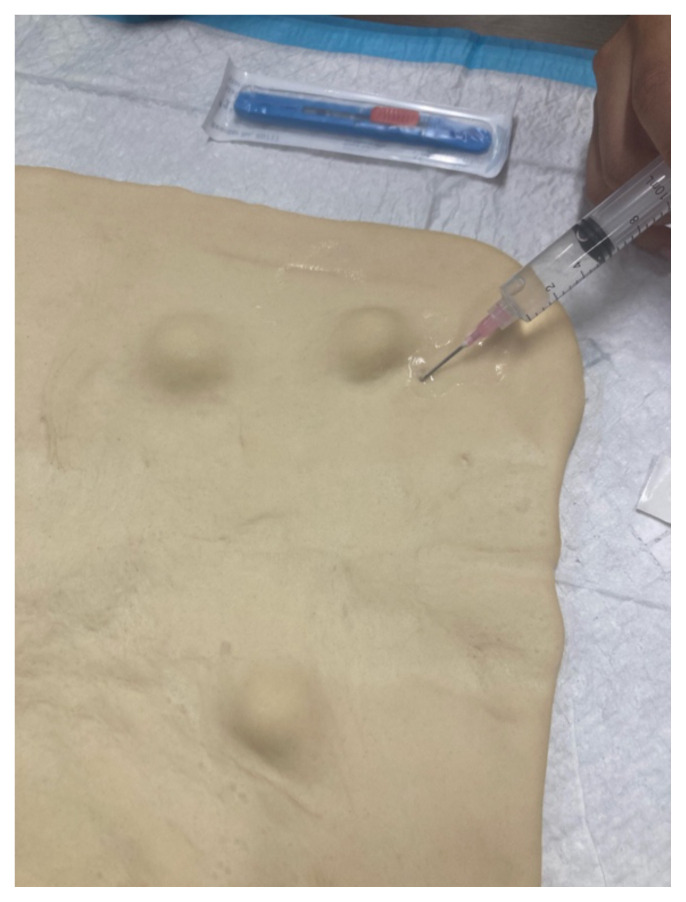
Demonstra7on of field block.

**Figure 3 f3-jetem-10-4-i7:**
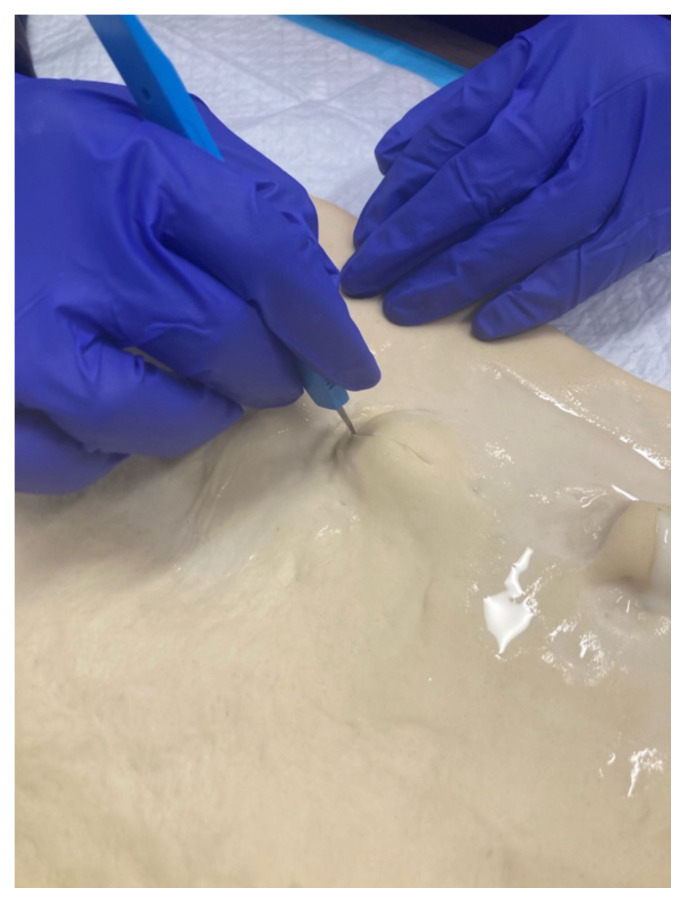
Incision into paintball.

**Figure 4 f4-jetem-10-4-i7:**
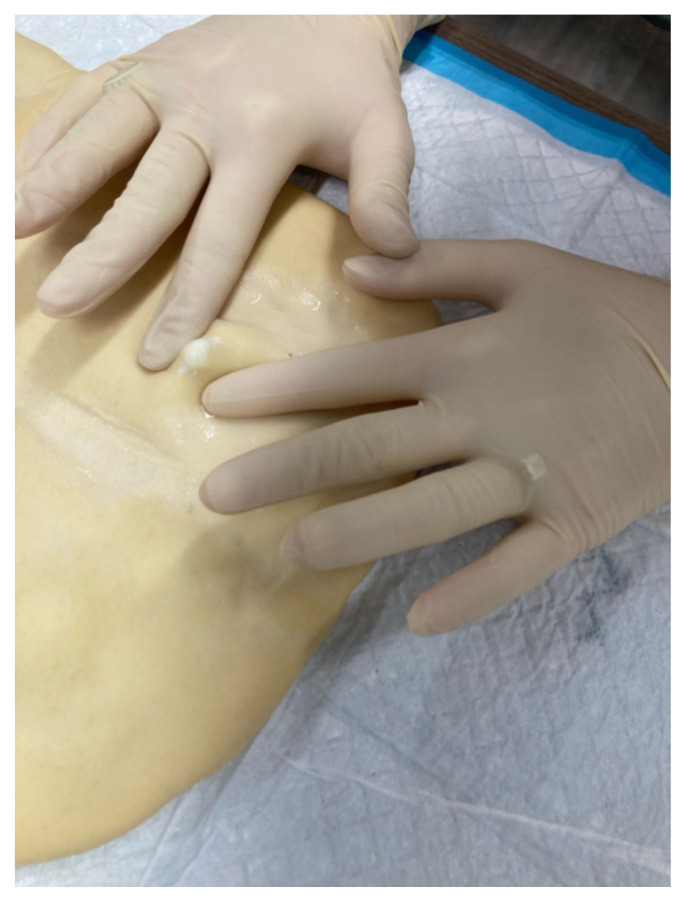
Expression of simulated purulent fluid.

**Figure 5 f5-jetem-10-4-i7:**
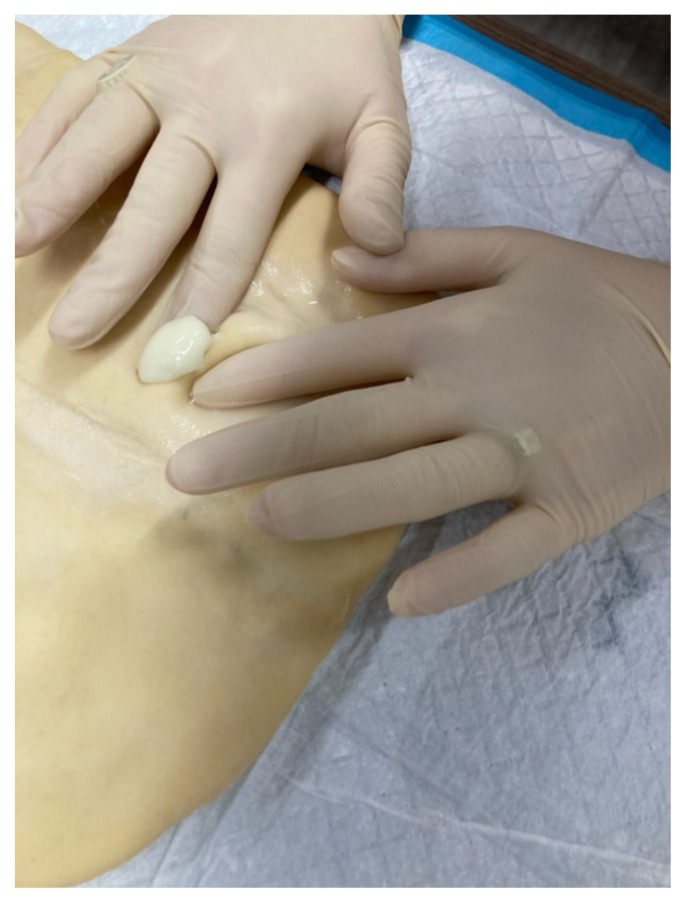
Manual expression of fluid.
