# Supplementary material for: Pizza and Paintballs: A Cost-Effective Model for Incision and Drainage Simulation Training
Source: J Educ Teach Emerg Med. 2025 Oct 31;10(4):I7–I15. doi: 10.21980/J8.52047 (PMC12594464; doi:10.21980/J8.52047)
Supplement: Supplementary file 1 [file 10-4-I7-Supp1.pptx]

## Slide 1
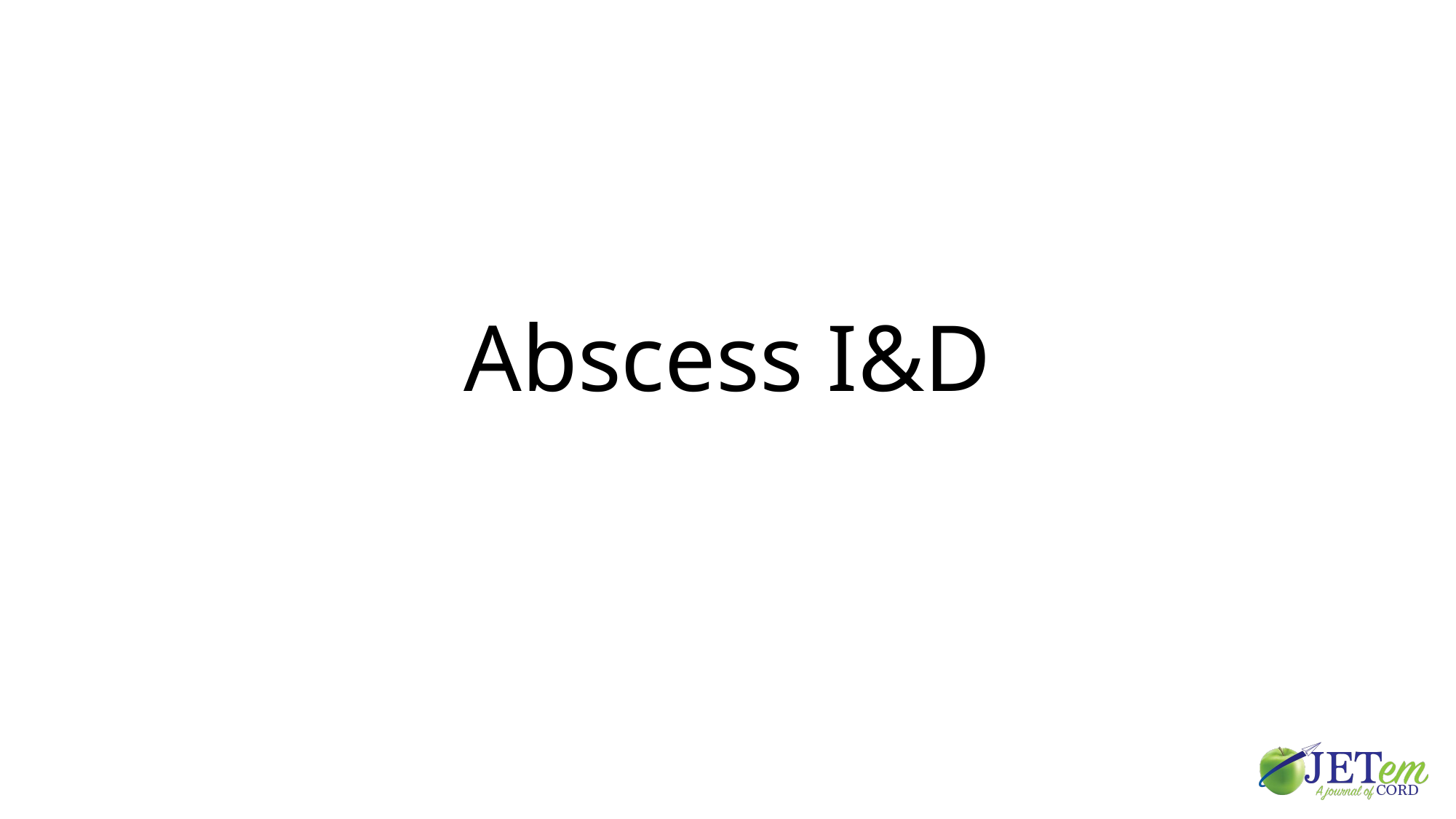

# Abscess I&D

## Slide 2
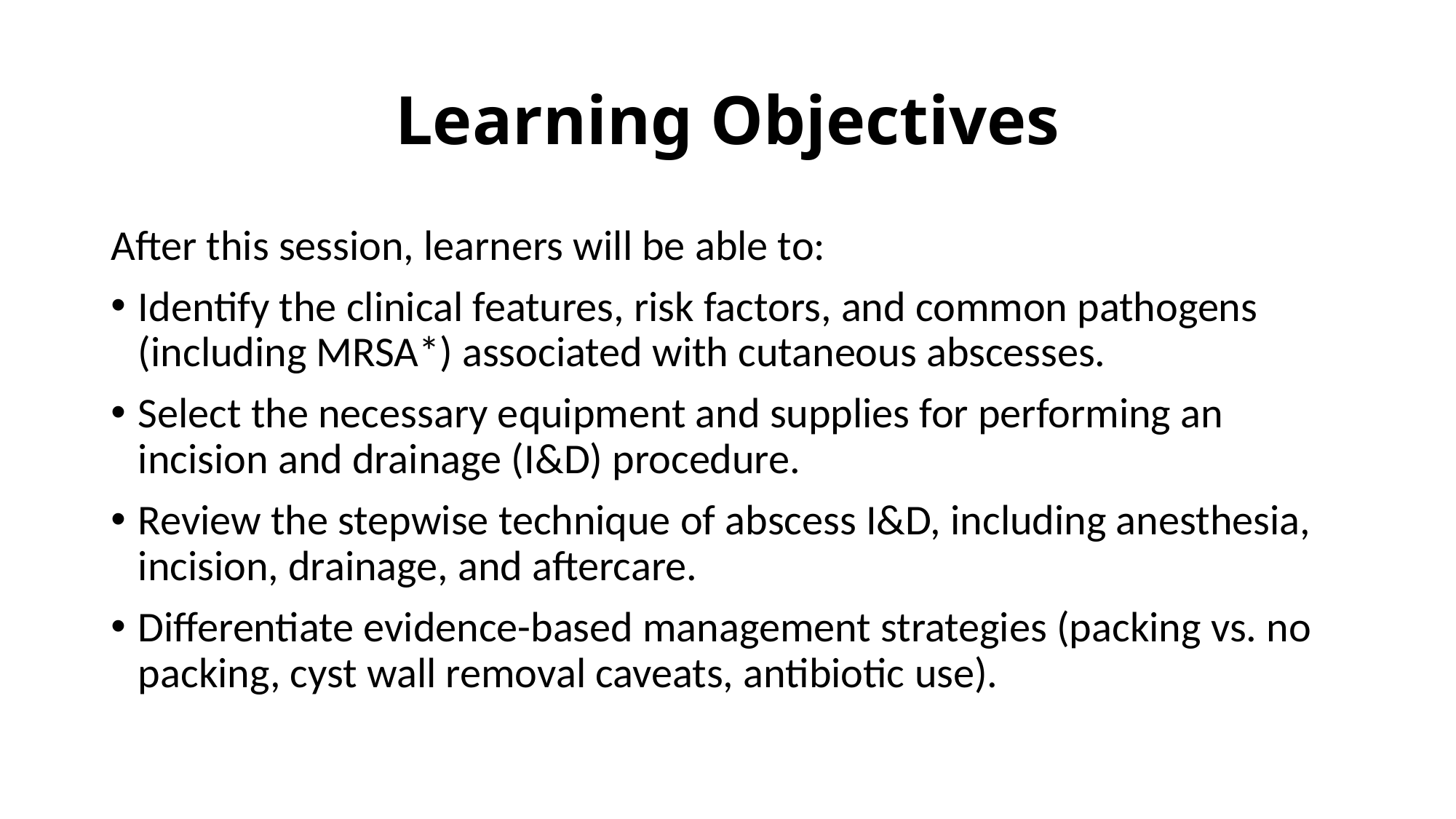

# Learning Objectives
After this session, learners will be able to:
Identify the clinical features, risk factors, and common pathogens (including MRSA*) associated with cutaneous abscesses.
Select the necessary equipment and supplies for performing an incision and drainage (I&D) procedure.
Review the stepwise technique of abscess I&D, including anesthesia, incision, drainage, and aftercare.
Differentiate evidence-based management strategies (packing vs. no packing, cyst wall removal caveats, antibiotic use).

## Slide 3
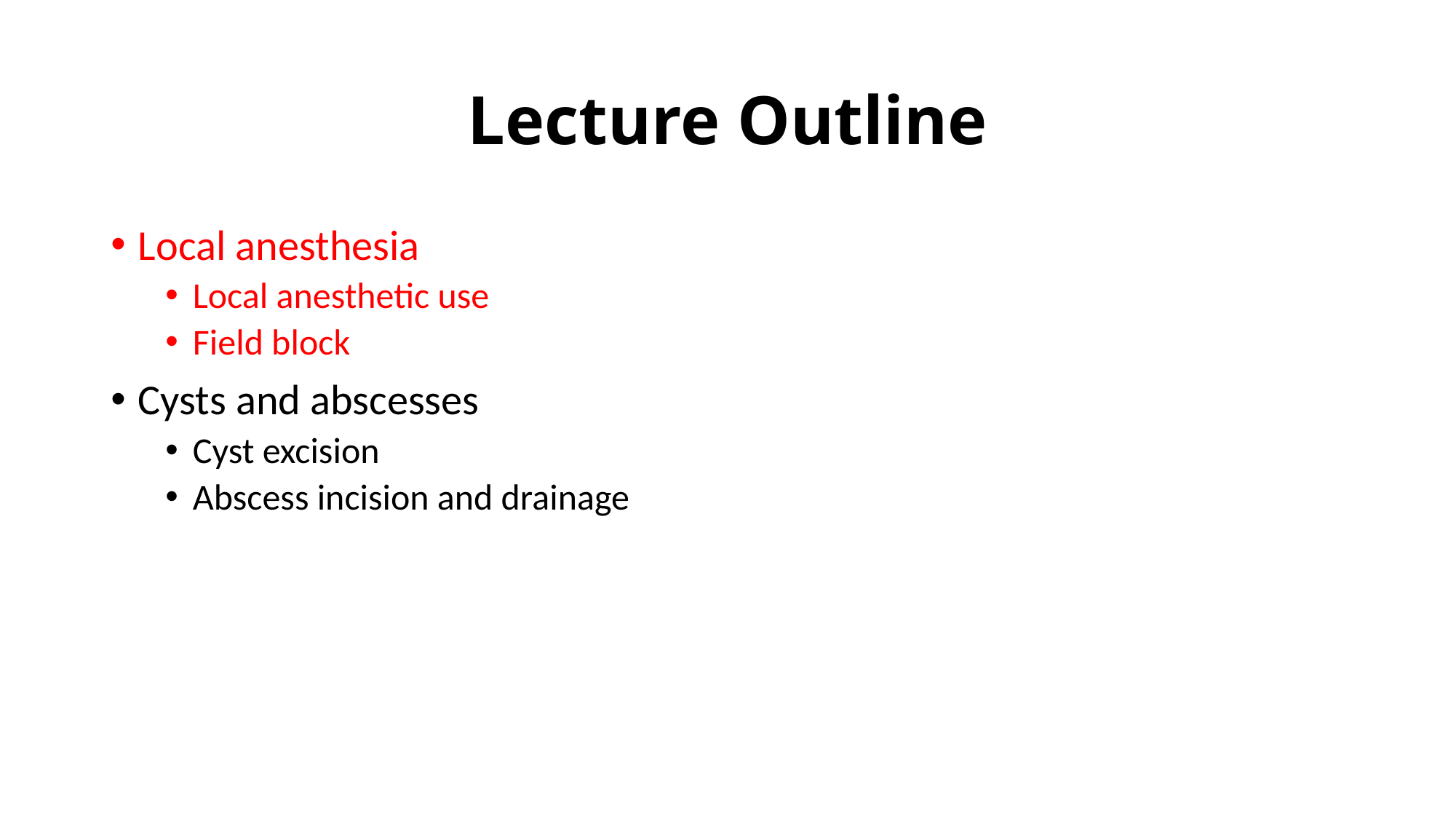

# Lecture Outline
Local anesthesia
Local anesthetic use
Field block
Cysts and abscesses
Cyst excision
Abscess incision and drainage

## Slide 4
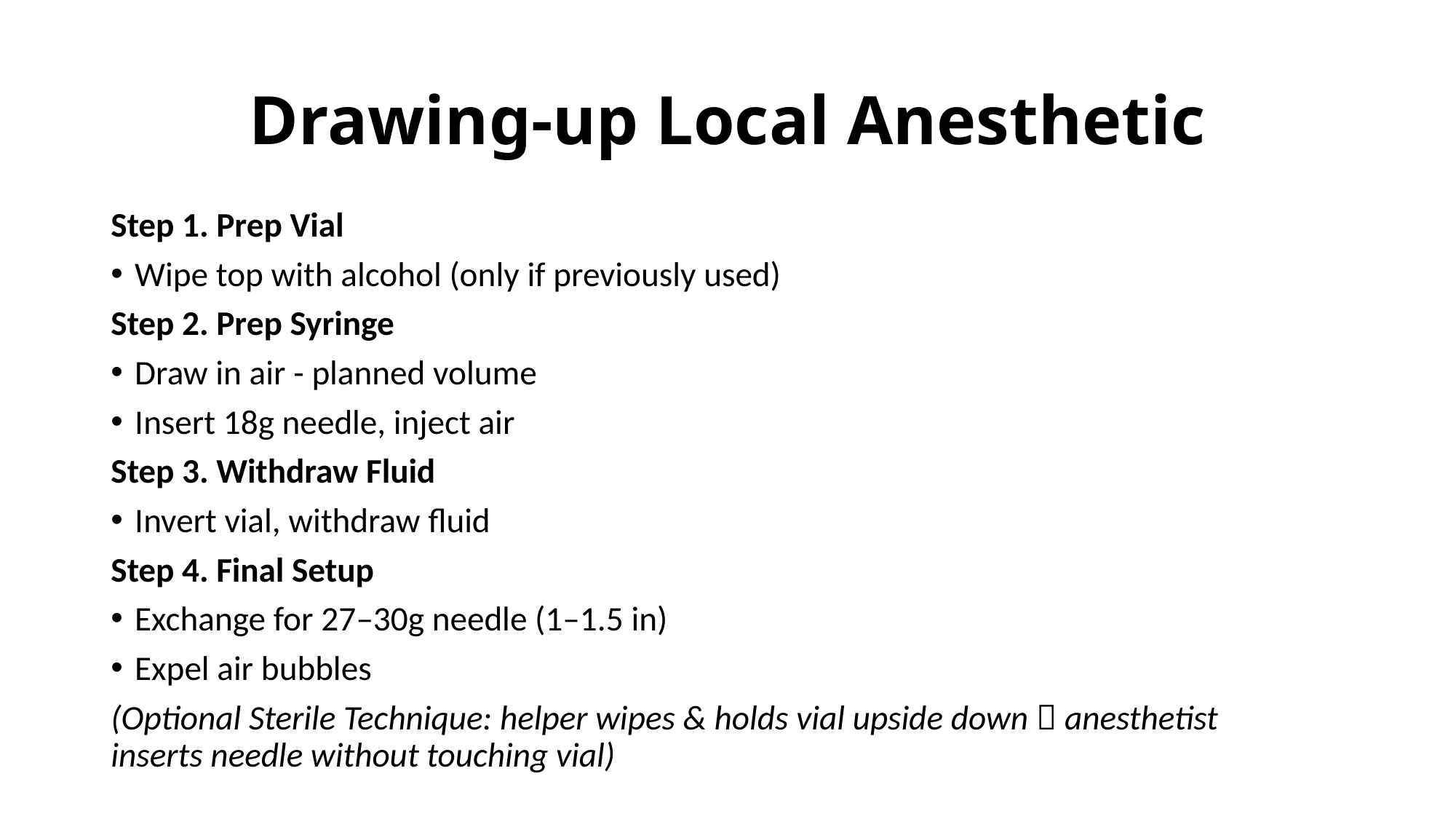

# Drawing-up Local Anesthetic
Step 1. Prep Vial
Wipe top with alcohol (only if previously used)
Step 2. Prep Syringe
Draw in air - planned volume
Insert 18g needle, inject air
Step 3. Withdraw Fluid
Invert vial, withdraw fluid
Step 4. Final Setup
Exchange for 27–30g needle (1–1.5 in)
Expel air bubbles
(Optional Sterile Technique: helper wipes & holds vial upside down  anesthetist inserts needle without touching vial)

## Slide 5
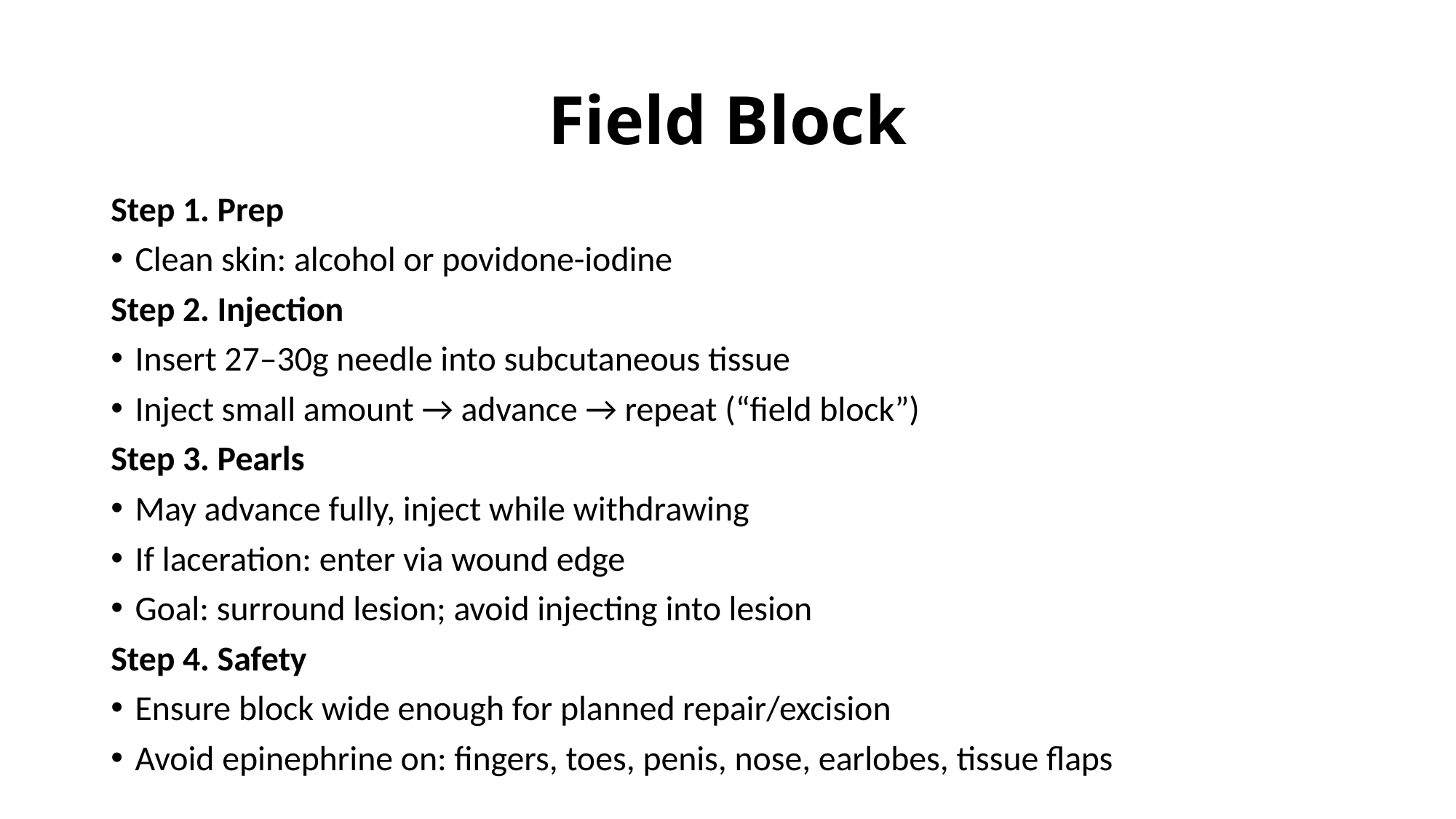

# Field Block
Step 1. Prep
Clean skin: alcohol or povidone-iodine
Step 2. Injection
Insert 27–30g needle into subcutaneous tissue
Inject small amount → advance → repeat (“field block”)
Step 3. Pearls
May advance fully, inject while withdrawing
If laceration: enter via wound edge
Goal: surround lesion; avoid injecting into lesion
Step 4. Safety
Ensure block wide enough for planned repair/excision
Avoid epinephrine on: fingers, toes, penis, nose, earlobes, tissue flaps

## Slide 6
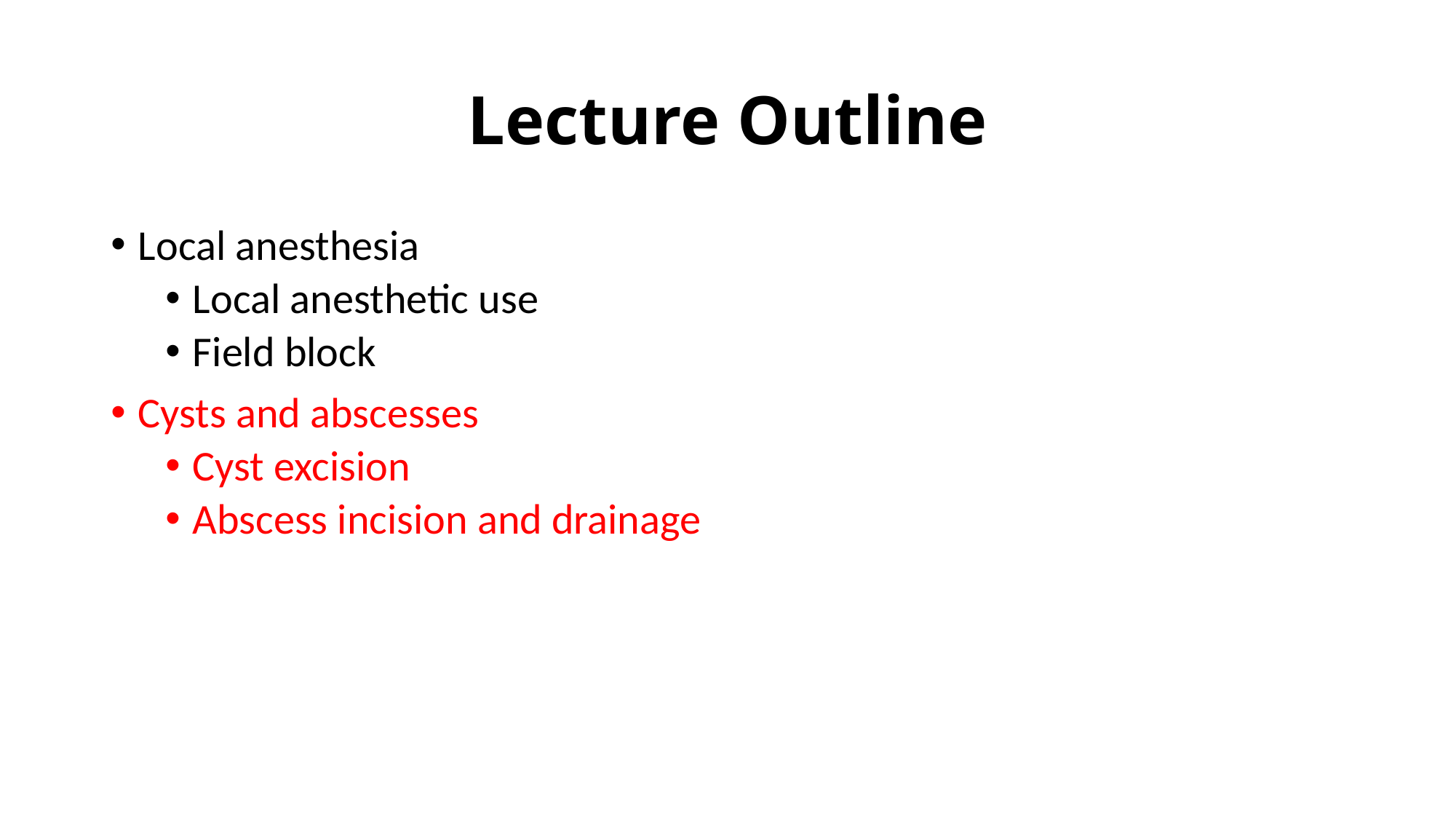

# Lecture Outline
Local anesthesia
Local anesthetic use
Field block
Cysts and abscesses
Cyst excision
Abscess incision and drainage

## Slide 7
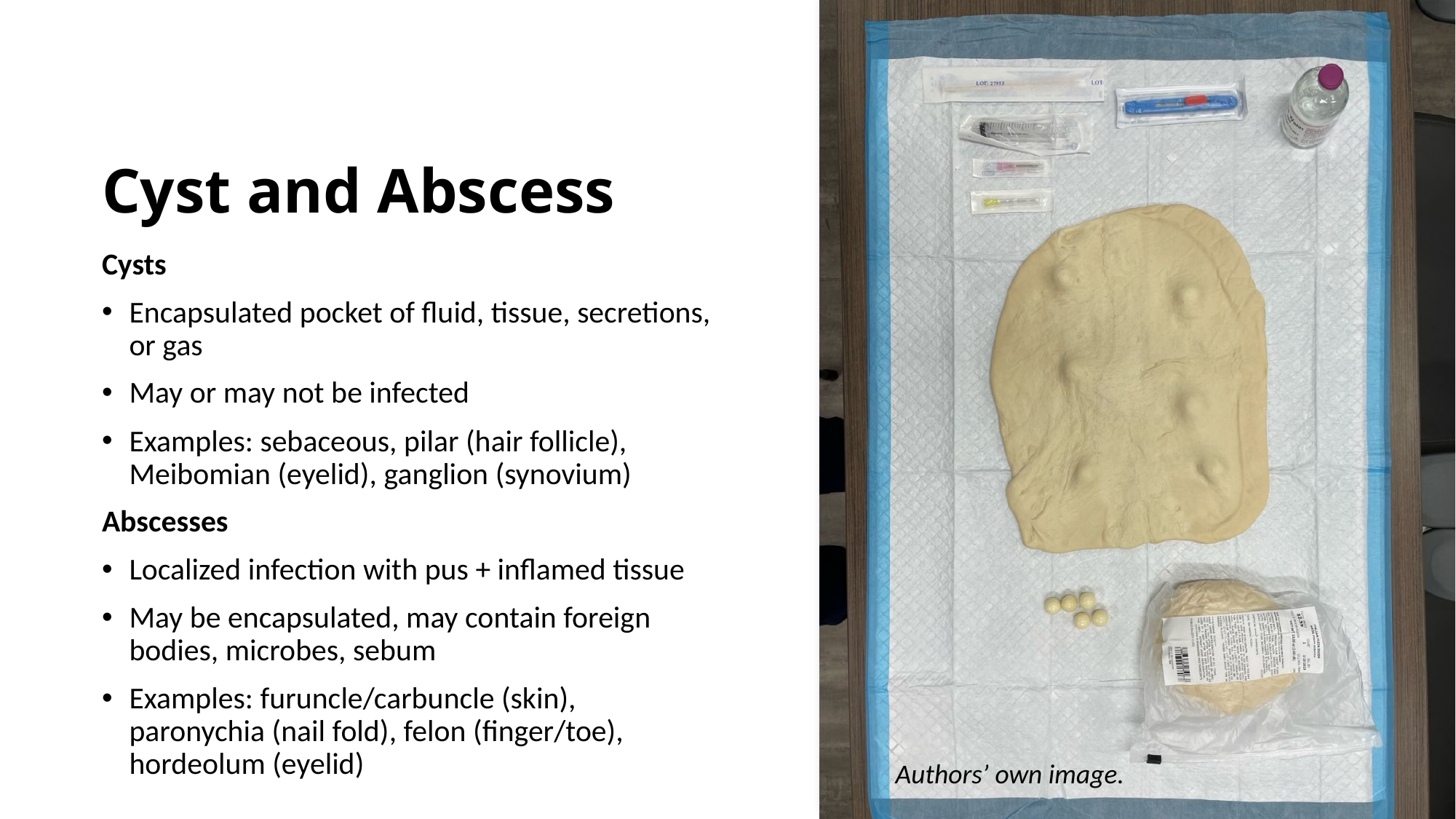

# Cyst and Abscess
Cysts
Encapsulated pocket of fluid, tissue, secretions, or gas
May or may not be infected
Examples: sebaceous, pilar (hair follicle), Meibomian (eyelid), ganglion (synovium)
Abscesses
Localized infection with pus + inflamed tissue
May be encapsulated, may contain foreign bodies, microbes, sebum
Examples: furuncle/carbuncle (skin), paronychia (nail fold), felon (finger/toe), hordeolum (eyelid)
Authors’ own image.

## Slide 8
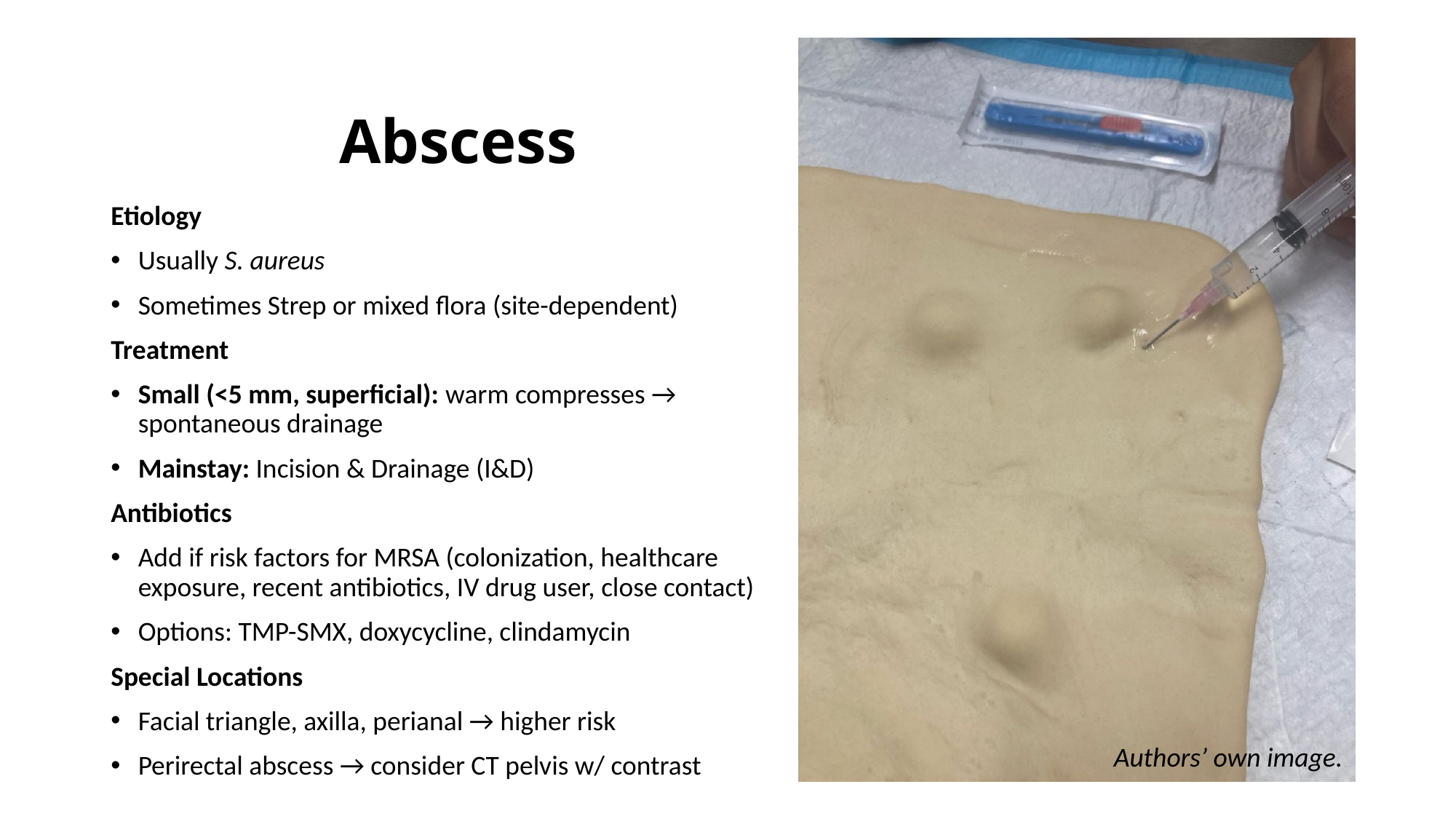

# Abscess
Etiology
Usually S. aureus
Sometimes Strep or mixed flora (site-dependent)
Treatment
Small (<5 mm, superficial): warm compresses → spontaneous drainage
Mainstay: Incision & Drainage (I&D)
Antibiotics
Add if risk factors for MRSA (colonization, healthcare exposure, recent antibiotics, IV drug user, close contact)
Options: TMP-SMX, doxycycline, clindamycin
Special Locations
Facial triangle, axilla, perianal → higher risk
Perirectal abscess → consider CT pelvis w/ contrast
Authors’ own image.

## Slide 9
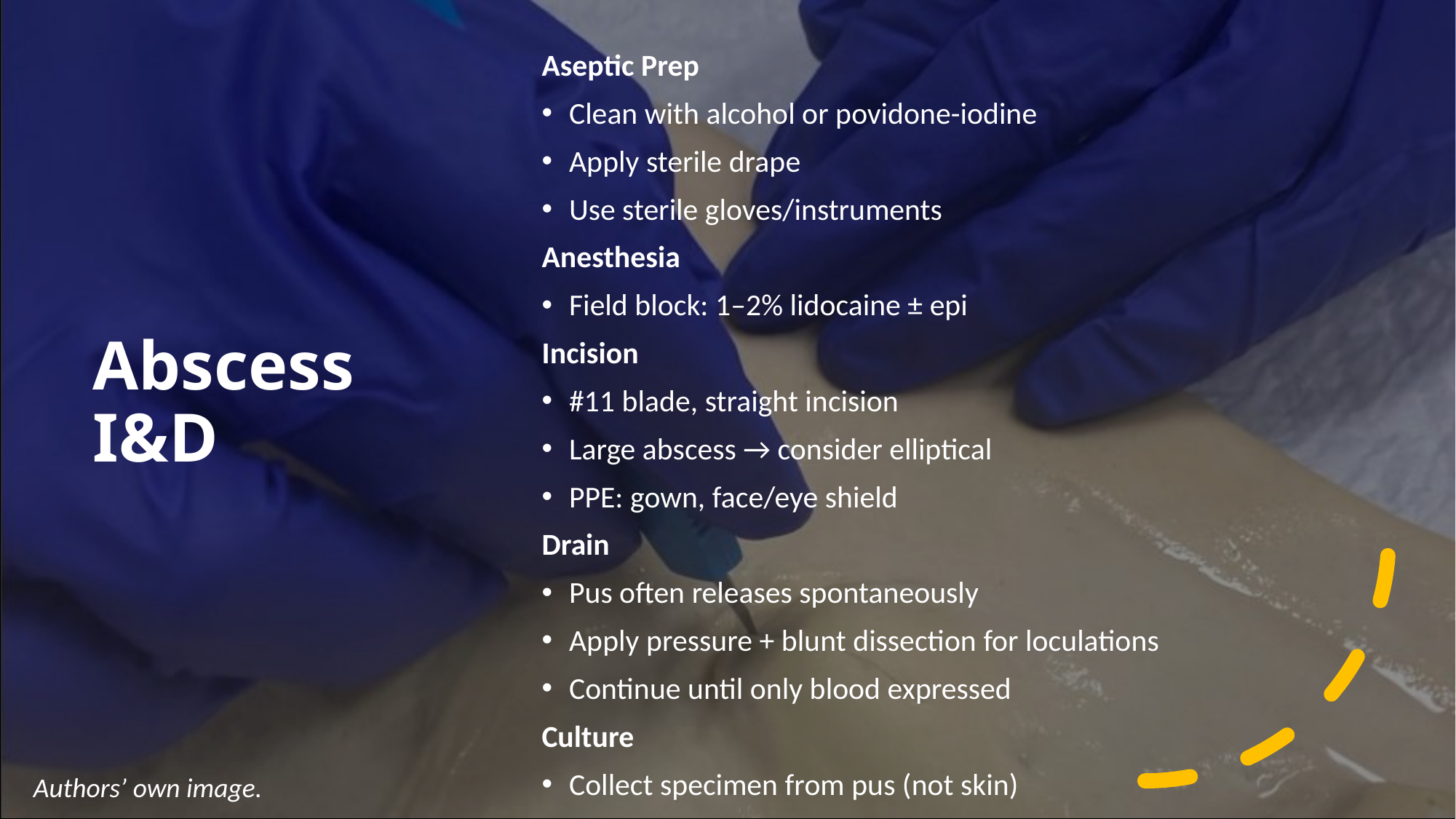

# Abscess I&D
Aseptic Prep
Clean with alcohol or povidone-iodine
Apply sterile drape
Use sterile gloves/instruments
Anesthesia
Field block: 1–2% lidocaine ± epi
Incision
#11 blade, straight incision
Large abscess → consider elliptical
PPE: gown, face/eye shield
Drain
Pus often releases spontaneously
Apply pressure + blunt dissection for loculations
Continue until only blood expressed
Culture
Collect specimen from pus (not skin)
Authors’ own image.

## Slide 10
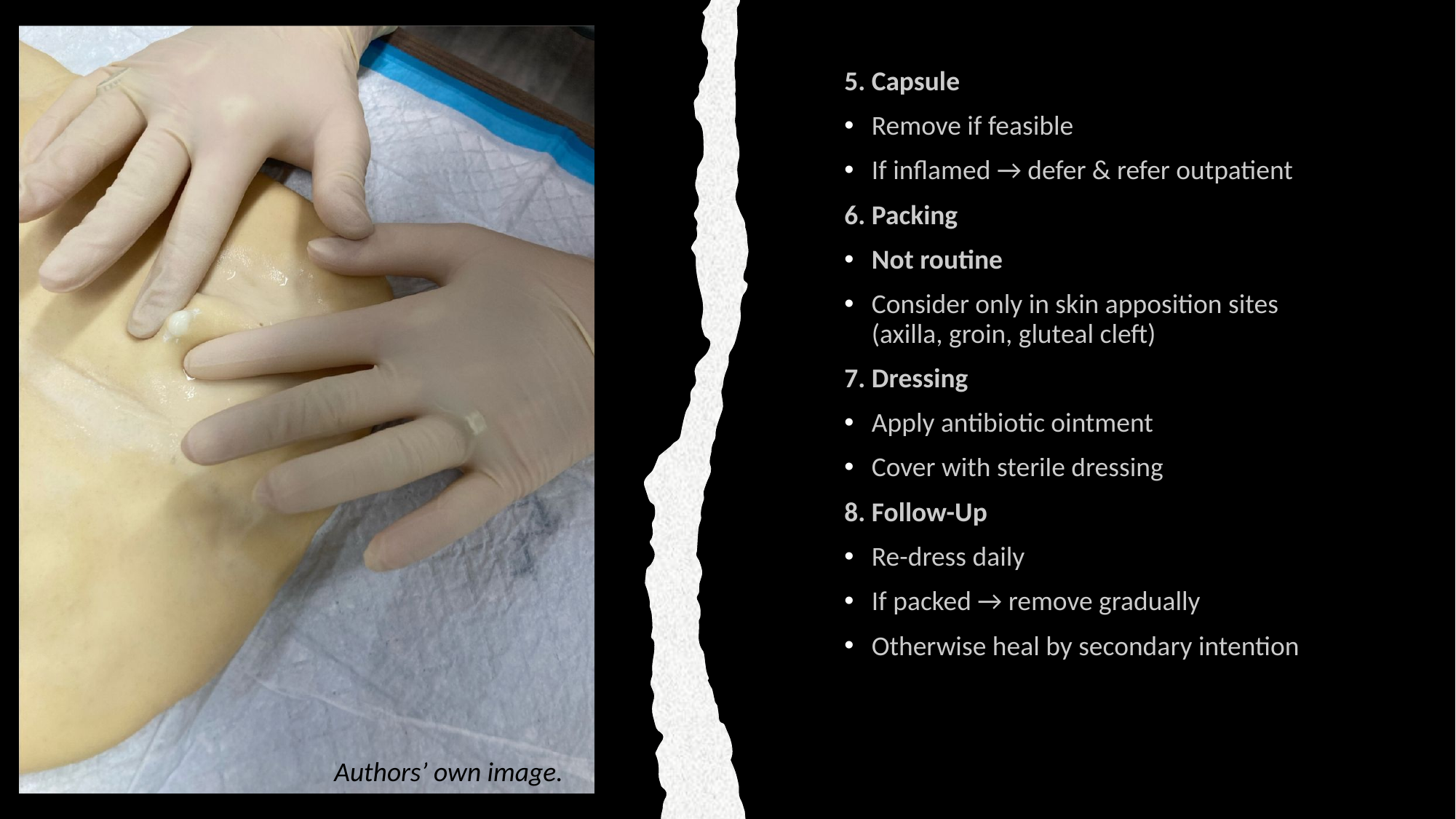

#
5. Capsule
Remove if feasible
If inflamed → defer & refer outpatient
6. Packing
Not routine
Consider only in skin apposition sites (axilla, groin, gluteal cleft)
7. Dressing
Apply antibiotic ointment
Cover with sterile dressing
8. Follow-Up
Re-dress daily
If packed → remove gradually
Otherwise heal by secondary intention
Authors’ own image.

## Slide 11
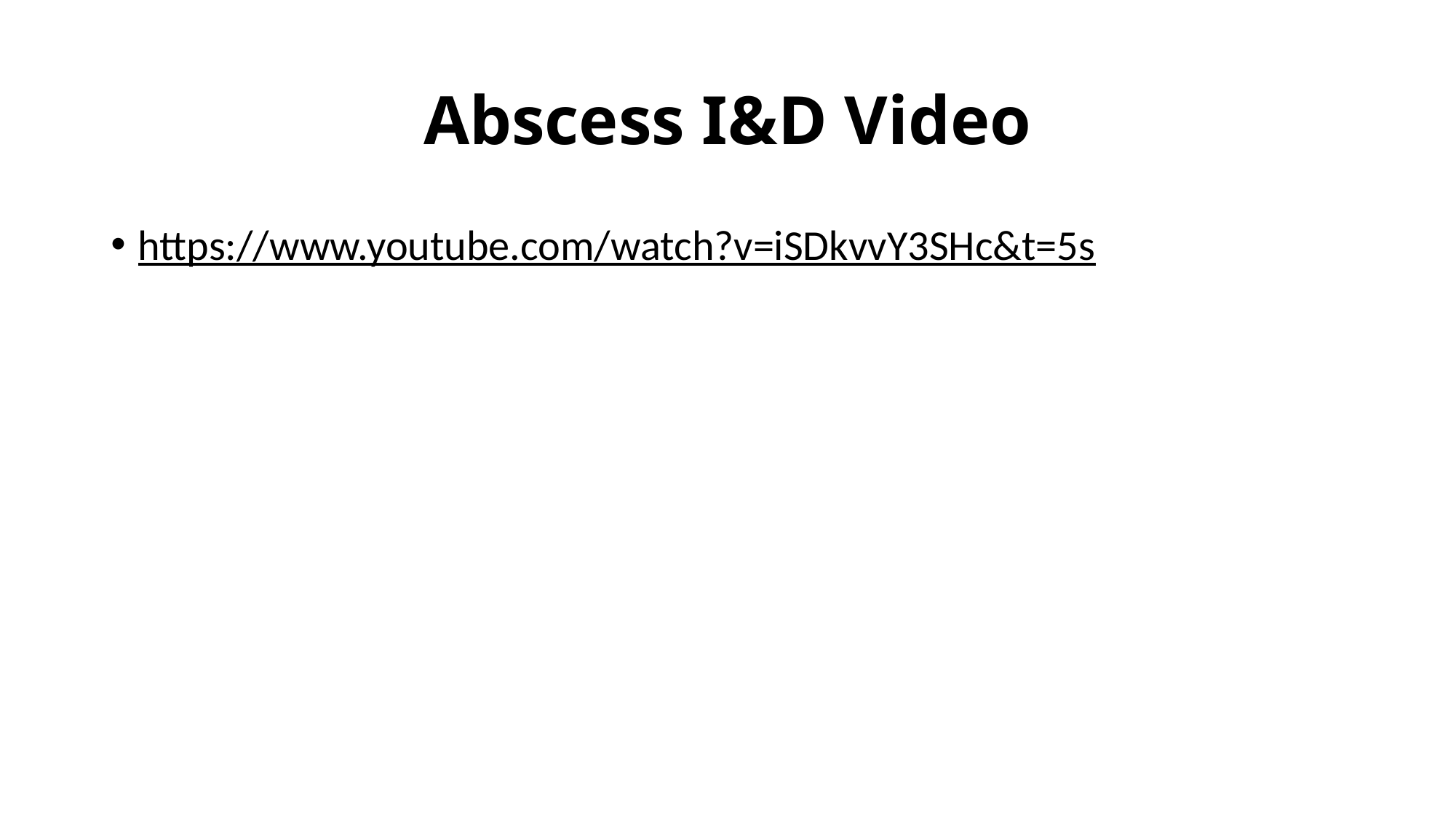

# Abscess I&D Video
https://www.youtube.com/watch?v=iSDkvvY3SHc&t=5s

## Slide 12
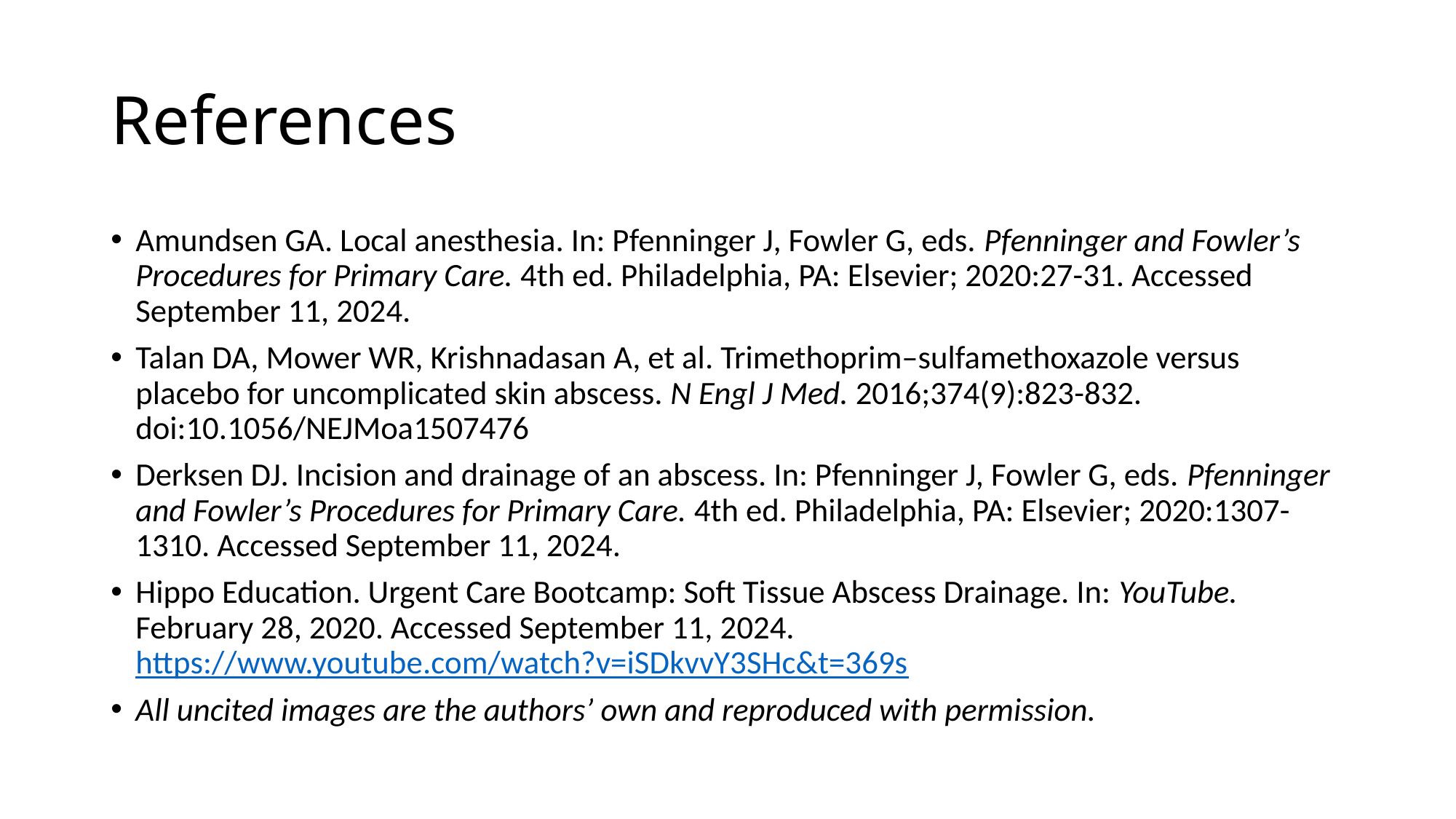

# References
Amundsen GA. Local anesthesia. In: Pfenninger J, Fowler G, eds. Pfenninger and Fowler’s Procedures for Primary Care. 4th ed. Philadelphia, PA: Elsevier; 2020:27-31. Accessed September 11, 2024.
Talan DA, Mower WR, Krishnadasan A, et al. Trimethoprim–sulfamethoxazole versus placebo for uncomplicated skin abscess. N Engl J Med. 2016;374(9):823-832. doi:10.1056/NEJMoa1507476
Derksen DJ. Incision and drainage of an abscess. In: Pfenninger J, Fowler G, eds. Pfenninger and Fowler’s Procedures for Primary Care. 4th ed. Philadelphia, PA: Elsevier; 2020:1307-1310. Accessed September 11, 2024.
Hippo Education. Urgent Care Bootcamp: Soft Tissue Abscess Drainage. In: YouTube. February 28, 2020. Accessed September 11, 2024. https://www.youtube.com/watch?v=iSDkvvY3SHc&t=369s
All uncited images are the authors’ own and reproduced with permission.
